# Pyoderma Gangrenosum in the Absence of Any Underlying Predisposing Condition: A Diagnostic Dilemma

**DOI:** 10.7759/cureus.4213

**Published:** 2019-03-09

**Authors:** Akriti G Jain, Mohamad Sharbatji, Ali Afzal, Summia Matin Afridi, Dwayne Gordon

**Affiliations:** 1 Internal Medicine, Florida Hospital, Orlando, USA

**Keywords:** pyoderma, ulcer, steroids, topical corticosteroids, non-infectious, auto-inflammatory disorder

## Abstract

Pyoderma gangrenosum (PG) is a rare non-infectious skin disease of undetermined origin. It is characterized by a single or multiple painful, necrotic ulcers. Formerly, PG was assumed to be infectious, but eventually, it was established to be an inflammatory disorder that is commonly associated with autoimmune and neoplastic diseases. We report a case of PG in a 70-year-old female who presented on the pretibial area as a single non-healing ulcer. It started as a small induration that worsened over the course of two weeks despite being on antibiotics. We started the patient on corticosteroids and high potency topical steroids that resulted in healing of the ulcer. PG can prove to be a diagnostic dilemma and can be inappropriately treated with antibiotics or even something radical like an amputation if misdiagnosed. Hence, physicians need to think of this entity even in the absence of any predisposing conditions.

## Introduction

Pyoderma gangrenosum (PG) as a term was first introduced by Brunsting et al. in 1930. They studied five patients having intractable ulcers; four of those patients had underlying ulcerative colitis and one had empyema [1]. At that time, PG was considered to be an infectious entity. But now it is believed to be an inflammatory disease characterized by a single or multiple painful destructive skin ulcers with a chronic relapsing course that heal with a scar [2]. PG is a rare disease and is estimated to affect three to 10 people in a million. It is commonly seen in adults of 30- to 60-year-age group, especially women [3].

PG is an idiopathic disease and its pathophysiology is still not completely understood. It is considered to be a neutrophilic dermatosis as a result of chemotaxis and up-regulation of polymorphonuclear neutrophils (PMNs). Although PG can occur anywhere on the body, ulcers are commonly seen on lower extremities (especially at the pretibial area) and trunk [4]. Typically the ulcer of PG begins as a small pustule that worsens rapidly over the course of days to weeks and progresses to a necrotic ulcer with undetermined borders [5]. PG ulcers often depict pathergy i.e. exacerbation of skin lesions following minor trauma, seen in 30% cases [6-7]. PG has clinical features similar to an infectious disease. We report a case of PG in a 70-year-old female without any underlying autoimmune or neoplastic disease.

## Case presentation

A 70-year-old female presented to our office with localized left leg swelling of two days duration after taking diclofenac for hip pain. The patient had a past medical history of hypertension, diabetes mellitus, asthma, osteoarthritis, and iron-deficiency anemia. She denied a history of any underlying autoimmune disease or inflammatory bowel disease. Her left lower extremity was red and had a localized fluctuant swelling of 2 x 2 cm with surrounding cellulitis. This was thought to be an abscess; incision and drainage (I and D) were performed on this visit. The patient was given amoxicillin-clavulanate and doxycycline with follow-up in one week.

On the next appointment, the abscess had worsened despite the antibiotics. The abscess was again opened and drainage was collected for culture and gram stain. On this visit, the patient’s antibiotics were changed to trimethoprim-sulfamethoxazole for one week. Cultures from this I and D were negative for any organism and so was the gram stain.

**Figure 1:**
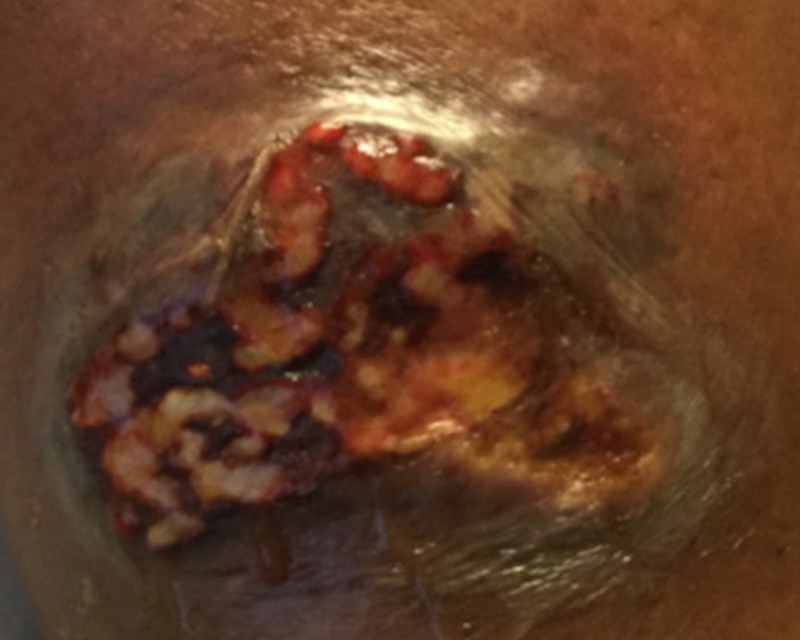
About 4.6-cm lesion on the pretibial area of the left lower extremity with undermined borders and denuded tissue and areas of necrosis as the base Mild to moderate serous fluid drainage was observed with a surrounding area of erythema.

**Figure 2:**
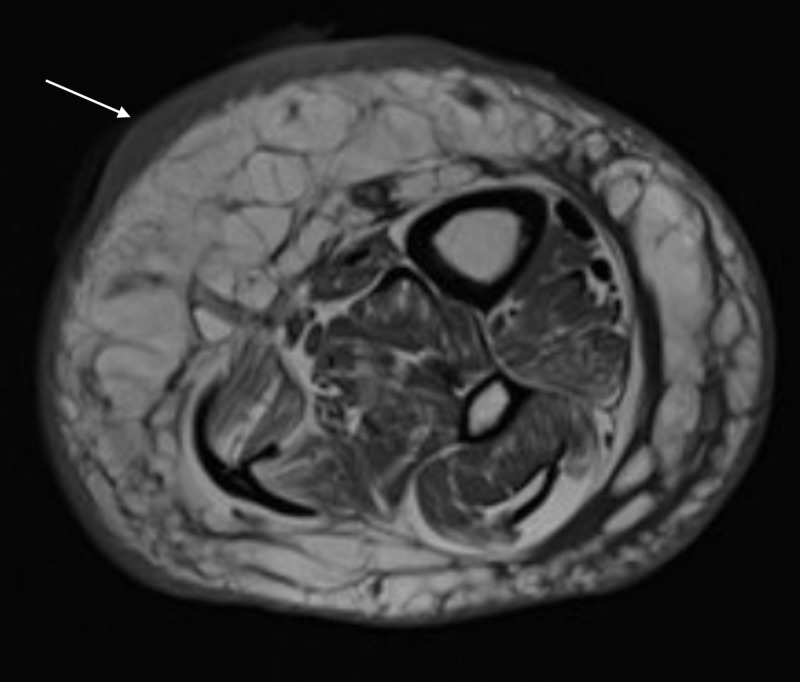
MRI showing wound (white arrow) medially in the distal calf with adjacent skin thickening along with circumferential subcutaneous edema MRI: magnetic resonance imaging

**Figure 3:**
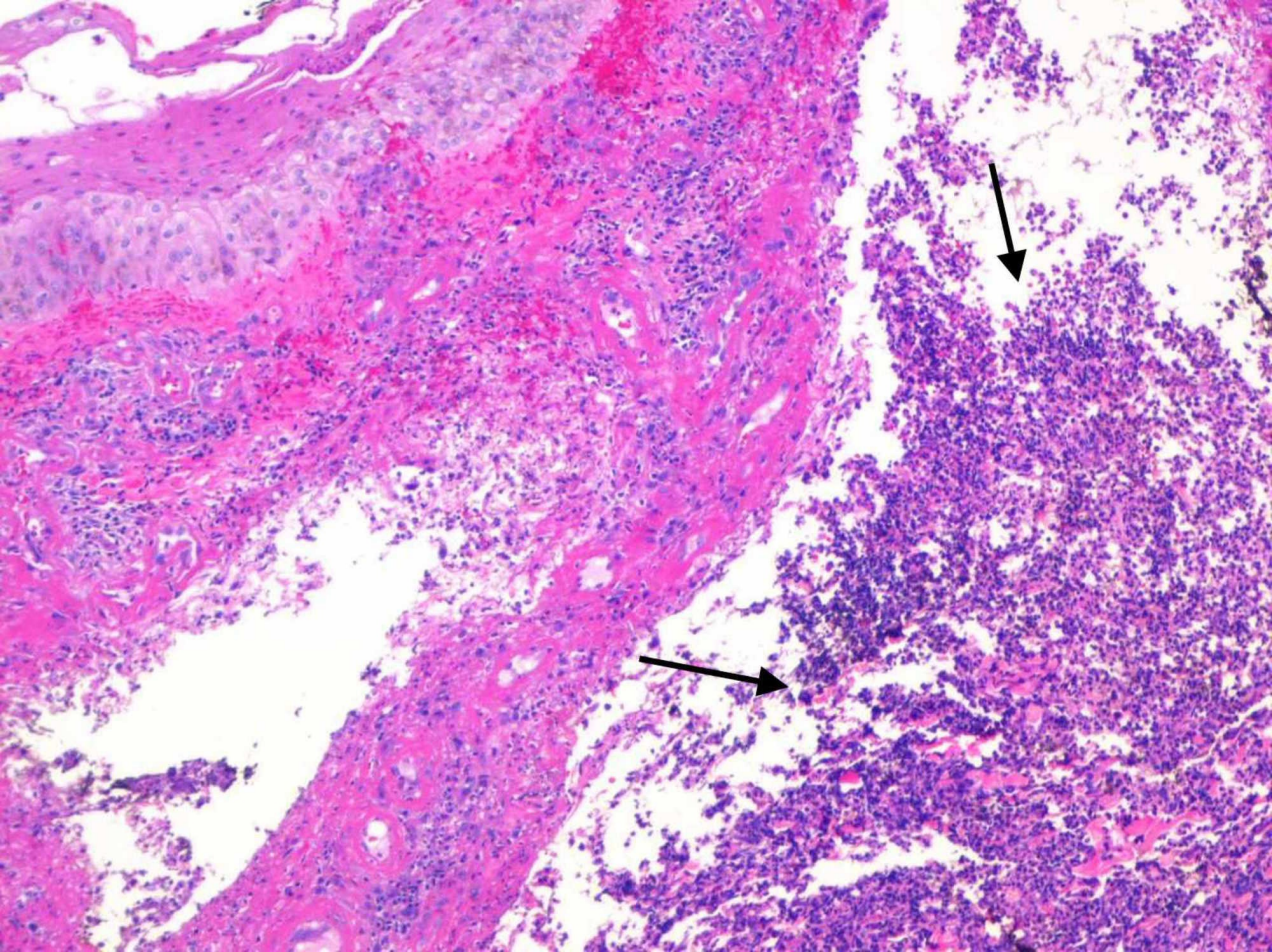
Pathology from punch biopsy of skin showing severe acute cellulitis, abscess formation, and granulation tissue (black arrows)

On the third visit, one week later, the wound had enlarged and was open (Figure 1). At this point, the patient was admitted to the hospital for further evaluation of a non-healing ulcer. On examination, the patient was afebrile and there was a 4.6-cm lesion on the pre-tibial area of the left lower extremity with undermined borders and denuded tissue and areas of necrosis as the base. Mild to moderate serous fluid drainage was seen with the surrounding area of erythema. Laboratory evaluation revealed the white blood cell count to be 9.28 x 10^3^/mL. MRI of the affected leg was done and it showed no evidence of osteomyelitis (Figure 2). Dermatology was consulted and punch biopsy was obtained. A sample of 0.3 x 0.3 cm was excised with a depth of 0.4 cm, and the sample was sent for microbiologic and pathologic analysis. On pathologic analysis of the sample (Figure 3), it was reported as severe acute cellulitis, abscess formation and granulation tissue which could potentially represent PG if infectious etiology can be completely ruled out. Microbiological analysis was negative for acid-fast bacilli, gram stain, or periodic acid-Schiff stain.

On the basis of two negative cultures, failure to respond to antibiotics, and pathologic analysis, the diagnosis of PG was established. The patient was started on oral prednisone 100 mg and high-potency topical steroids. After a couple of days, there was a marked reduction in erythema and ulcer showed signs of healing. A workup for secondary causes including recent EGD and colonoscopy was negative for malignancy and inflammatory bowel disease, and there was no evidence of any inflammatory or rheumatologic condition. On follow-up at one week after hospital discharge, the wound was found to be improving.

## Discussion

In the 1930s, PG was considered to be a rare skin disease caused by a bacterial infection in an immunocompromised host [1]. It derives its name from its misdiagnosis as a purulent streptococcal infection (pyoderma) causing necrosis of tissue (gangrenosum). Fulbright et al., in 1985 hypothesized that PG is due to an abnormal immune response with unidentified underlying factors [8]. But the misnomer “pyoderma gangrenosum” is still used in medicine due to its typical clinical manifestations.

Approximately 50% of all cases of PG have underlying autoimmune diseases like inflammatory bowel disease, vasculitis, Sjogren’s disease, ankylosing spondylitis, systemic lupus erythematosus, myeloproliferative, or myelodysplastic disorders [9-10]. In our patient, no underlying condition was known to be associated with PG. In 2013, Pereira N et al. in their review of 24 cases, reported two cases of PG which were not associated with any underlying disease [11]. There are four variants of PG including classical or ulcerative, pustular, bullous, and vegetative or superficial granulomatous [12]. The variant of PG that we observed in our patient was ulcerative or classic, which is characterized by rapidly progressive ulcers having undetermined edges and surrounding erythema. 

PG is a diagnosis of exclusion and it is especially hard for physicians to diagnose PG because blood cultures are usually negative and ulcers are sterile as seen in our case. The diagnosis of PG is based on the typical natural history of the disease and the lack of response to antibiotics [13]. Classically, the ulcer is described as a lesion that creeps centrifugally with central oozing, undermined violaceous necrotic borders and peripheral erythema. Healing occurs with the formation of a central cribriform atrophic scar and significant disfiguration. Ulcers of PG can occur anywhere on the body, but the ulcers of the classic or ulcerative variant of PG usually occur on the pretibial area [14]. Our patient also presented with a pretibial ulcer and it was categorized into the classic variant of PG based on the physical appearance and examination. Other areas where PG has been seen include hands, abdomen, head and neck, genital, and in atypical cases around the peristomal region, surgical sites and internal sites like kidney and lungs [7]. Histopathological features of PG are not specific. It is a neutrophilic dermatosis of undetermined significance. Neutrophils are the cytological hallmark of PG [4,11].

The other differential diagnoses considered in our case were skin and soft tissue infection (SSTI) and drug-induced PG as our patient developed the ulcer two days after taking diclofenac. SSTI was ruled out because the patient did not respond to antibiotics [15]. We concluded that the diagnosis was not drug-induced PG since the ulcer worsened even after holding diclofenac and Naranjo score was way low to support this differential.

Treatment of PG can be very hard especially in a non-dermatological setting because PG is misdiagnosed as an infectious disease and treated according to infectious disease guidelines using antibiotics that can worsen the condition rather than improving it as seen in our case [16]. In a review, Haag et al. found 38 cases of PG which were misdiagnosed out of which amputation was considered in 14 cases and complete or partial amputation was performed in six cases with penile amputation in one case proving the importance of correct diagnosis of PG [17]. There is no gold standard therapy for PG and currently, the mainstay of therapy is to target the immunologic mediators and inflammatory cells using topical and systemic corticosteroids [18]. Although PG begins to improve within a few days of therapy with corticosteroids, it requires weeks to months to achieve complete healing of the ulcer [19]. Other treatment options include topical calcineurin inhibitors such as cyclosporine and tacrolimus. In cases of severe and generalized disease steroid-sparing agents including cyclophosphamide, azathioprine, methotrexate, and cyclosporine can be used [7]. Optimized wound care is imperative in the treatment of PG in the form of moist sterile dressings and wound cleansing.

## Conclusions

The learning point we inferred from our case is that PG can occur even in the absence of underlying autoimmune or neoplastic disease and clinicians should think of PG even when cultures are negative for infection and histology shows neutrophilic predominance in the absence of infection. Misdiagnosis can result in catastrophic outcomes and early as well as appropriate treatment with corticosteroids plays a critical role in the outcome of this disease. 
